# Effect of humic acid derived from leonardite on the redistribution of uranium fractions in soil

**DOI:** 10.7717/peerj.14162

**Published:** 2022-10-07

**Authors:** Fande Meng, Qiuxiang Huang, Yongbing Cai, Guodong Yuan, Liang Xiao, Fengxiang X. Han

**Affiliations:** 1 College of Resource and Environment, Anhui Science and Technology University, Chuzhou, Anhui Province, China; 2 Department of Chemistry, Physics and Atmospheric Sciences, Jackson State University, Jackson, MS, United States of America; 3 Guangdong Provincial Key Laboratory of Environmental Health and Land Resource, Zhaoqing, Guangdong Province, China

**Keywords:** Humic acid, Soil pH, Redistribution, Dynamic behavior, Uranium fraction

## Abstract

Humic acids (HAs) are complex organic substances with abundant functional groups (*e.g.*, carboxyl, phenolic-OH, *etc*.). They are commonly distributed in the soil environment and exert a double-edged sword effect in controlling the migration and transformation of uranium. However, the effects of HAs on dynamic processes associated with uranium transformation are still unclear. In this study, we used HAs derived from leonardite (L-HA) and commercial HA (C-HA) as exogenous organic matter and C-HA as the reference. UO_2_, UO_3_, and UO_2_(NO_3_)_2_ were used as the sources of U to explore the fractionations of uranium in the soil. We also studied the behavior of the HA. The incubation experiments were designed to investigate the effects of HA on the soil pH, uranium fraction transformation, dynamic behavior of exchangeable, weak acid, and labile uranium. The observations were made for one month. The results showed that soil pH decreased for L-HA but increased for C-HA. Under these conditions, uranium tended to transform into an inactive fraction. The dynamic behavior of exchangeable, weak acid, and labile uranium varied with the sources of HA and uranium. This study highlighted that HA could affect soil pH and the dynamic redistribution of U fractions. The results suggest that the sources of HA and U should be considered when using HA as the remediation material for uranium-contaminated soils.

## Introduction

Uranium (U) is one of the most dangerous heavy metals and is radioactive. It is present in abundance in the earth’s crust, and the average crust content of U is 2.6 mg/kg (Rankin, 2008). Most rocks typically contain 0.4–3.7 ppm of U (Escareño Juárez et al., 2019). In nature, there are three isotopes of uranium: U^238^ (99.275%), U^235^ (0.72%), and U^234^ (0.054%), and depleted uranium (DU) usually contains 0.3% of U^235^ (or less). The content of U in the soils varies from 0.7 to 10.7 mg/kg (Kabata-Pendias & Pendias, 1985). Studies conducted on the U content in the soils revealed that the differences in the content are related to the soil’s texture, and it does not depend on the soil type: the U content was low (0.3 mg/kg) in coarse soils and high (11 mg/kg) in fine soils (Shacklette & Boerngen, 1984). The very high concentrations (even more than ten thousand mg/kg) are attributed to the technogenic contamination of soils, and the contaminations can be contributed by weapons testing, scientific research, nuclear power station accident, and U mining (Kazery et al., 2021). Generally, the common U valences in the soil are IV or VI. U(IV) can transform into U(VI) under oxidative conditions (Gu et al., 2005). U can be transferred into the human body through food chains, surface water, and groundwater (Dinocourt et al., 2015). It can cause risks to human health and various cause damage to the reproductive system. Neurotoxicology, liver and kidney toxicity, and increased possibility of cancer can also be attributed to the presence of U (Dinocourt et al., 2015).

Depleted uranium (DU) is produced from enriched-U with lower U^235^, which is used in military and civilian applications (used for developing armor-piercer and radiation shields) (Betti, 2003). The staff is directly affected by U pollution. The use of DU increases the content of uranium-containing wastes that are released into the soil environments. The U in soil participates in redox reactions and interacts with soil components, influencing the U transformation behavior in soil (Chen et al., 2021). Soil organic matter, one of the most important soil components, plays a key role in controlling the mobility and bioavailability of uranium. Gu et al. (2005) studied natural humic substances and observed that humic acids (HAs) affect the process of uranium reduction and oxidation to influence its mobility. Bednar et al. (2007) found that the complexation of organic matter with uranium affects its redistribution. HAs are the main components of soil organic matter, and these significantly affect the process of transformation of heavy metals in soil environments (Wei et al., 2007; Meng et al., 2021a).

HAs are widely distributed in the soil environment. These are key factors that control the processes of migration and transformation of uranium (Sachs & Bernhard, 2011). The HAs are complexes of organic matter. These are soluble in alkali but form precipitate with acids. The HAs contain abundant functional groups, especially acidic groups (carboxylic and phenolic-OH), to adsorb uranium ions. Meng et al. (2021b) found the HAs derived from leonardite exhibited high adsorption capacity even at a low pH, and the carboxylic was the main group for adsorption of U(VI). The interaction of HAs with U plays a role in controlling the migration and transformation of U in the soil environments (Sachs & Bernhard, 2011). Zhang et al. (2020) reported that the presence of HAs can reduce the mobility of U(VI), while Bednar et al. (2007) reported that the presence of HAs resulted in a decrease in the uranium content in soils. Bednar et al. (2007) also reported that in the presence of HAs, the potential desorption ability of uranium increased. In addition, HAs can act as electron providers or electron acceptors to induce a valence change and affect uranium distribution in soil environments. Gu et al. (2005) reported that the natural humic substances could exert bioreduction and oxidation effects on uranium. Wang et al. (2019) explored the abiotic reduction of U (VI) in the presence of HAs. Wang et al. (2019) reported that in the presence of HAs, highly soluble U(VI) could be transformed into insoluble U(IV). Therefore, HAs exert significant effects on the migration and transformation behavior of U present in soil environments.

Numerous researchers have studied the effects of HAs on U in soils (Gu et al., 2005; Bednar et al., 2007; Wang et al., 2019; Zhang et al., 2020). However, the effects of HAs on the dynamic behavior of U obtained from various sources are still unclear. In this study, U(IV), U(VI), and HA derived from leonardite were used for the incubation experiments. Commercial HA was used for control comparison. Meng et al. (2021b) revealed the HA derived from leonardite exhibited high adsorption affinity for U(VI). The objectives of this paper were (1) to explore the effect of HA on the dynamic behavior of uranium fraction and (2) to investigate the effect of HA on the redistribution of U in soil. The results can help understand the mechanism following which HAs affect uranium present in the soil environments.

## Materials & Methods

### Chemical reagents and humic acid

The leonardite was obtained from the Shandong Chuangxin Humic Acid Technology Co., Ltd., Shandong Province, China. The traditional alkaline–acid extraction method was used to extract HA (L-HA) (Meng et al., 2021b), and the physical and chemical properties were analyzed. The pH was determined by a pH meter (Mettler Toledo, Switzerland) in distilled water at a ratio of 1:10 (g:mL). The ash content was determined based on the weight of L-HA before and after calcination in a muffle furnace at 800 °C (time:4 h), and the experiments were conducted under atmosphere conditions. The organic carbon content was determined using an elemental analyzer (Vario micro cube; Elementar, Langenselbold, Germany), and the samples were dried at 85 °C until a constant weight was obtained. Fourier transform infrared (FTIR, Nicolet IS10; Thermo Fisher, Waltham, MA, USA) was used to analyze functional groups, and the carboxyl group content was determined following the titration method outlined by the International Humic Substances Society (IHSS, 2022). A commercial HA (C-HA, pH 10.20) with high solubility (almost 20% percent of total at a ratio of 1:10 (g:mL)) was used as the reference material, and it was purchased from Alfa Aesar, USA.

The used chemicals and reagents were of guaranteed reagent (GR) grade. Ca(NO_3_)_2_, NaHCO_3_, and Na_2_CO_3_, were purchased from Thermo Fisher Scientific, Waltham, MA, USA. UO_2_ and UO_3_ were obtained from the International Bio-Analytical Industries Inc., Boca Raton, FL, USA, and UO_2_(NO_3_)_2_ was purchased from Poly scientific R&D Corp., Bay Shore, NY, USA.

### Soil sampling, experimental design, and analysis

Fresh surface paddy soil (0–30 cm) was collected from the Mississippi River Delta in the USA. The soil samples were air-dried at room temperature and then sieved through a 2-mm mesh and thoroughly mixed. UO_2_, UO_3_, and UO_2_(NO_3_)_2_ were used as the sources of U. One U content, 100 mg/kg, the level was applied. U treatments include control, and 60 mg/kg U mixed with 600 g of the soil. C-HA and L-HA were used as exogenous organic matters to influence the uranium fractions. Two HA contents (calculated by C) were mixed with the soil, and the HA content was varied as 0 (control sample), 1000, and 3000 mg C/kg. UO_2_, UO_2_ and UO_2_(NO_3_)_2_ and HA was mixed with air-dried soil to ensure homogeneity, respectively. The mixtures of soil, U, and HA were incubated under 60% field water capacity at room temperature for one month.

The soil samples were analyzed for the following properties: (1) pH in distilled water (1:5 w/v ratio; determined using a pH meter (Oakton, USA)); (2) organic carbon (analyzed using an elemental analyzer (Elemental combustion system; Costech, Valencia, CA, USA)); (3) pseudo total U concentration by placing 0.5 g of soil (air-dried) into a polytetrafluoroethylene vessel. A 3HCl:1HNO_3_ (v/v) mixture (four mL) was added, and the mixture was heated in a microwave oven (*t* = 90 °C). After cooling, the extracts were filtered through a 0.45 mm membrane into 50 mL glass flasks. The flasks were filled to the mark with ultra-pure water. The samples were then analyzed using the inductively coupled plasma mass spectrometry (ICP-MS) (820-MS; Varian, Inc., Valencia, CA, USA) technique

### Extraction and analysis

#### Extraction of exchangeable, weak acid-soluble, and labile U

The non-sequential selective method of exchangeable, weak-acid soluble and labile U were applied to reflect the direct and potential mobility/toxicity of U in soil environment. The extracting solutions for exchangeable, weak acid-soluble, and labile U were prepared as described by Smith et al. (2009). Ca(NO_3_)_2_ (0.5 M; pH 5.09) targets the extraction of exchangeable U, 0.44 M CH_3_COOH + 0.1 M Ca(NO_3_)_2_ (pH 2.75) targets the extraction of weak-acid soluble U, and 0.014 M NaHCO_3_ + 0.0028 M Na_2_CO_3_ (pH 9.64) targets the extraction of labile U.

To study the dynamic behavior of HA on exchangeable, weak acid-soluble, and labile U, we sampled soil samples at 1, 3, 7, 12, 18, 24, and 30 days. The soil samples were air-dried under room temperature and subsequently ground through a 2-mm mesh for extraction. Briefly, 1 g (accurate 0.0001 g) of soil samples were taken into 15 mL tubes containing 10 mL of the extractants. The tubes were put in a temperature-controlled shaker at 25 °C for 16 h. After this, the tubes were centrifuged at 3,000 g for 15 min and filtered through a 0.45-µm membrane for U analysis (the ICP-MS technique was used).

#### U fractions: extraction and analysis

The sequential selective extraction method can provide the insight into the changes of the U bound to soil components, reflecting the effects of HA on the redistribution of U fractions in soil environments (Smith et al., 2009). The seven-step sequential selective extraction method was used to extract U. The U fractions were classified as exchangeable (EXC), bound to carbonate (CAR), bound to easily reducible oxides (ERO), bound to organic matter (OM), bound to amorphous iron oxides (AmoFe), bound to crystalline iron oxides (CryFe) and residual fractions (RES) and details of sequential selective extraction are presented in Meng et al. (2018).

### Data process

All extraction experiments were performed in duplicate. The tube was weighed before and after each step to determine the amount of the solution remaining in the residual soil. Quality assurance and quality control (QA/QC) were estimated using duplicates, and the standard deviation of duplicate samples was within 5%. In addition, a series of standards were run every 30 samples to calibrate the instruments during U analysis, and the results showed the instrument was in good condition and data were reliable/valid.

The data were collected and analyzed method as previously in Meng et al. (2018).

## Results & Discussion

### Properties of the soil samples and L-HA

The basic properties of soil are shown in Table 1. The soil was slightly alkaline (pH 7.57), and the organic carbon content was 1.16%. According to soil classification, the soil was silt clay (USDA, 2022). The actual U concentrations in the soil when the cultivation experiment was conducted with UO_2_, UO_3_, and UO_2_(NO_3_)_2_ as the sources of U were 84.6, 102.0, and 96.5 mg/kg, respectively. L-HA was characterized by a low pH of 4.02, and the ash content was 7.87%. The carbon content was as high as 58.7%, and with a low solubility (below 1% of total at a ratio of 1:10 (g:mL)). The FTIR showed that L-HA contains abundant functional groups, such as carboxyl, phenolic-OH, and amino, and the carboxyl content was 3.15 mol/kg, indicating that L-HA exhibited a high combing ability for cations (Shi et al., 2018).

**Table 1:** Basic properties of soil.

	pH	Organic Carbon (%)	Sandy (%)	Silt (%)	Clay (%)	Total U (mg/kg)
Paddy soil	7.50	1.16	2.00	42.00	56.00	0.77

### Effect of HA on soil pH and organic matter

**Figure 1:**
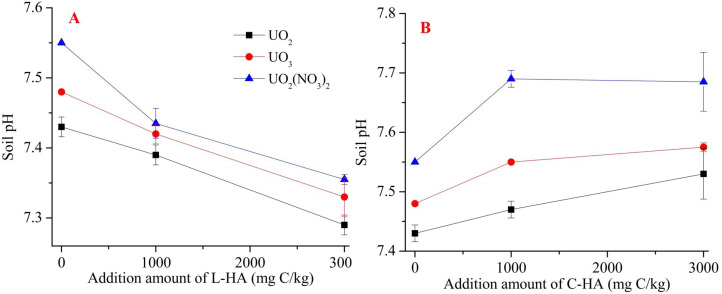
The effects of HA on soil pH after one month incubation. (A) L-HA, (B) C-HA.

The pH value is one of the basic soil properties which can directly influence the mobility and bioavailability of the metals. It can affect the toxicity of heavy metals in the soil (Houben, Evrard & Sonnet, 2013; Król, Mizerna & Bozym, 2020). Heavy metal mobility has previously been reported to negatively affect soil pH. A low soil pH results in high mobility and bioavailability of heavy metal cations (Houben, Evrard & Sonnet, 2013; Król, Mizerna & Bozym, 2020). HA is characterized by high buffer capacity as it contains many functional groups. Therefore, it significantly affects the pH of the soil. Thus, adding HA to the soil causes changes in soil pH, and the extent of the changes realized is affected by the HA content, that the higher dosage of L-HA cause lower pH (Fig. 1A) and higher dosage of C-HA caused higher pH (Fig. 1B). Figure 1 reveals that L-HA helped decrease soil pH by 0.15 to 0.20, while C-HA helped increase soil pH by 0.1 to 0.15. This can be attributed to the fact that L-HA, which is characterized by a low pH (4.02), can consume alkaline materials at a low pH, and C-HA (characterized by a high pH (10.20)) can release alkaline materials into the soil, increasing the soil pH. This indicates that L-HA can potentially increase the mobility of U in soil (Houben, Evrard & Sonnet, 2013; Król, Mizerna & Bozym, 2020). Also, we found that the pH of the soil varied based on the sources of U. This can be attributed to the chemical processes with which U in the soil environments was associated (Meng et al., 2018).

Organic matter also is one of basic properties of soil, and it plays a key role in the process of detoxification of toxic materials present in soil environments. It also affects the mobility and the fraction of heavy metals in soils (Ziółkowska, 2015). It has been previously reported that soil organic matter (SOM), such as humic substances (HS), can influence the migration of metals, which affects the mobility and the heavy metal contents in soil (Zhou et al., 2018; Bone et al., 2019). We mixed HA with soil and observed that adding HA increased the SOM content, indicating that the mobility and fractionation of U in the soil can potentially be influenced (Zhou et al., 2018; Bone et al., 2019). C-HA with high pH was highly soluble, while L-HA with low pH was poorly soluble. This affects U mobility (Clemente & Bernal, 2006; Janoš et al., 2010). Thus, adding HA can influence the mobility and fractionation of metals by influencing soil pH and SOM content.

### Effect of HA on mobility and bioavailability of U in soil

**Figure 2:**
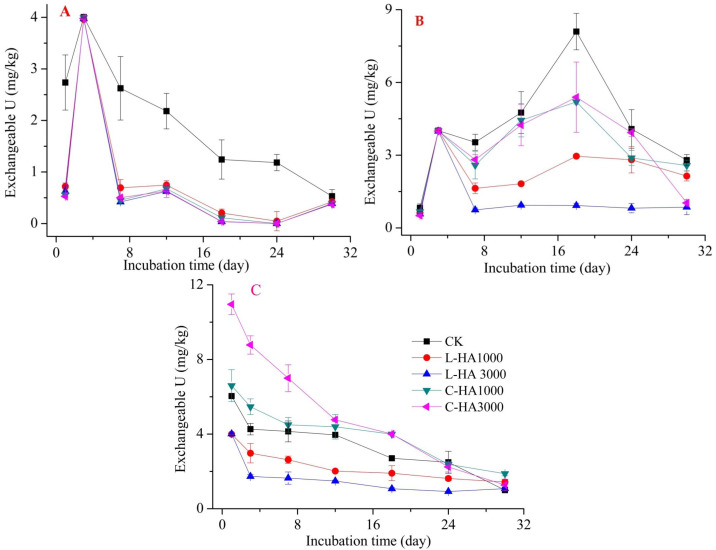
The effect of HA on the exchangeable U. (A) U_2_O, (B) UO_3_, (3) UO_2_(NO_3_)_2_.

**Figure 3:**
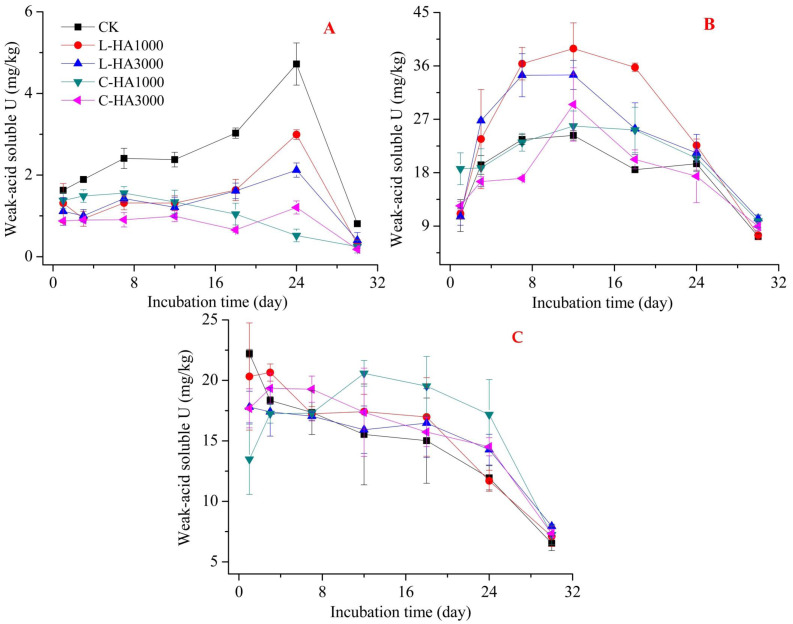
The effect of HA on the weak-acid soluble U. (A) U_2_O, (B) U_3_O, (C) UO_2_(NO_3_)_2_.

**Figure 4:**
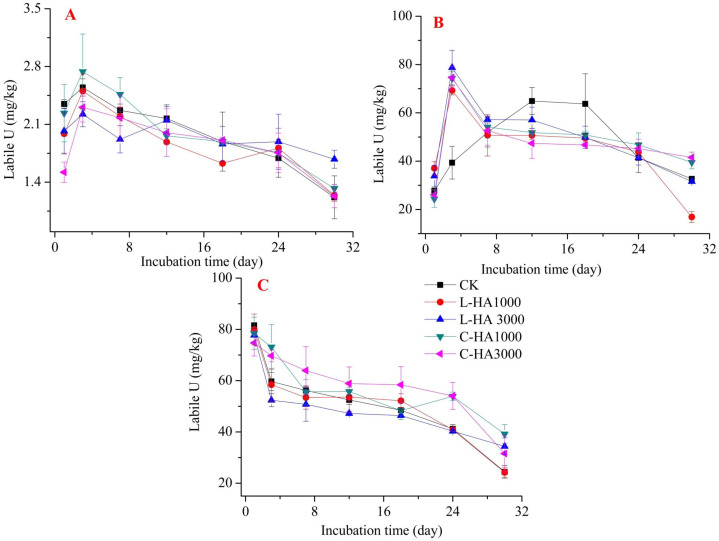
The effect of HA on the labile U. (A) UO_2_, (B) UO_3_, (C) UO_2_(NO_3_)_2_.

The mobility and bioavailability are strongly related to the fractionation of heavy metals. This can be affected by organic matter, such as HA (Hernandez-Soriano & Jimenez-Lopez, 2012; Zhang et al., 2020). Thus, adding HA into soil affects the redistribution of U in soil (Figs. 2, 3 and 4). In general, oxidation conditions are predominant in natural soil, and UO_2_ may become mobile and transform into U(VI) (Abdelouas, 2006). HA containing abundant functional groups not only can be considered as an electron-donator but also an electron-acceptor. It can transfer an electron from HA to Fe(III) and accept an electron from U(IV) to HA (Gu et al., 2005; Karimian, Burton & Johnston, 2019). On the other hand, HA can adsorb heavy metals, which can affect their dynamic behavior in soil (Karimian, Burton & Johnston, 2019; Zhang et al., 2020; Meng et al., 2022). Thus, the dynamic behavior of U in the soil can be influenced by HA in soil.

#### Effect of HA on the dynamic behavior of exchangeable U in soil

Exchangeable U in the soil can directly affect the mobility and toxicity of U. It can be uptaken by plants, soil animals, and microorganisms. The results showed that the addition of HAs significantly affected the dynamic behavior of exchangeable U. Analysis of Fig. 2 reveals that the dynamic behavior of exchangeable U differed based on the sources of U and HA. For UO_2_ and UO_3_ (Fig. 2A and 2B), the addition of L-HA and C-HA reduced the exchangeable U content, and this can be potentially attributed to the fact that the addition of HA can form complexes with soil cations, such as Ca^2+^ (Hering & Morel, 1988). HA can also participate in the abiotic reduction process to decrease the solubility of U (Wang et al., 2019). During the whole incubation process, the exchangeable U content increased during the first stage (0–3 days for UO_2_ and 0–18 days for UO_3_, respectively) and then decreased. Compared to CK, adding HA decreased the exchangeable U content, which can be potentially attributed to the fact that adding HA increased the adsorption capacity of the soils for cations (Piri, Sepehr & Rengel, 2019). For UO_2_(NO_3_)_2_ (Fig. 2C), the dynamic behavior of the exchangeable U content decreased during the incubation period. The addition of L-HA resulted in a decrease, but the addition of C-HA resulted in an increase in the content. We also found that the content of exchangeable U varied with addition dosage of HA. The higher the dosage of L-HA, the lower the content of exchangeable U, and the higher the dosage of C-HA, the higher the exchangeable U content. This can be potentially attributed to the differences in the L-HA and C-HA contents and the resources of U. UO_2_(NO_3_)_2_ is a total ionization chemical agent in soil. Unlike UO_2_, which is characterized by low solubility in soil environments, UO_3_ needs time to form UO_2_^2+^ in soil, causing a decrease in the exchangeable U of UO_2_(NO_3_)_2_ content in the soil environment during the incubation time (Meng et al., 2018). L-HA is an insoluble HA with abundant functional groups. It can increase the number of adsorption sites in soils for UO_2_^2+^. C-HA, characterized by high pH and solubility, increases the soil pH to increase the dissolved organic matter in the soil. This indicates that a large amount of UO_2_^2+^ was extracted as exchangeable U by Ca(NO_2_)_2_. The amount extracted was larger than the amount of L-HA extracted (Borggaard, Holm & Strobel, 2019).

#### Effect of HA on the dynamic behavior of weak-acid soluble U in soil

Weak acid U can be easily released into the soil–water system and can be taken up by plants and microorganisms such as exchangeable U. The addition of HA affects the properties of the weak-acid soluble U present in the soil. However, the effects were different for UO_2_, UO_3_, and UO_2_(NO_3_)_2_. The difference in effects can be attributed to the difference in the sources of U and HA (Fig. 3). The addition of L-HA and C-HA exerted adverse effects on soil pH. L-HA decreased soil pH while C-HA increased the soil pH. This can have different effects on the properties of weak-acid-soluble U. For UO_2_ (Fig. 3A), the addition of HA reduced the weak acid soluble U content, which may be due to the adsorption of U onto HA. For UO_3_ and UO_2_(NO_3_)_2_ (Fig. 3B and 3C), the addition of HA showed adverse effects, indicating that the addition of HA increased the weak acid soluble U content. This can be attributed to the fact that the addition of L-HA resulted in a decrease in the soil pH, and the addition of C-HA resulted in an increase in the soil dissolved organic matter content. This resulted in an increase in the weak acid soluble U content (Borggaard, Holm & Strobel, 2019; Król, Mizerna & Bozym, 2020). Also, we found that the weak acid soluble U content was influenced by the dosage of HA. For UO_2_, the higher the HA content, the lower the weak acid soluble U content. For UO_3_ and UO_2_(NO_3_)_2_, the lower the dosage of HA, the higher the weak acid soluble U content.

#### Effect of HA on the dynamic behavior of labile U in soil

Labile U can reflect the potential bioavailability and toxicity of U. The addition of HA has different effects on the labile U contents, and the effect varies based on the sources of U and HA (Fig. 4). When UO_2_ and UO_3_ are the sources of U, during the first phase (Fig. 4A and 4B), the labile U content increased sharply, and at this stage, HA played a role in enhancing the content. As the time increased, the labile U content decreased. However, the content increased with the addition of HA, indicating that HA can promote the formation of active insoluble U (Gu et al., 2005). A similar effect was observed when the source was UO_2_(NO_3_)_2_ (Fig. 4C). Thus, the addition of HA increased the potential bioavailability and toxicity of U, resulting in the high solubility and pH of HA that caused U to transform to labile U.

Compared to the exchangeable U and weak-acid soluble U content, the labile U content is significantly higher. Several factors could cause this, such as: (1) increased pH. HA can form complexes with U, and HA is soluble in bases but insoluble in acids. This results in an increase in the concentration of labile U; (2) U cations can easily combine with carbonate agents (Santos & Ladeira, 2011). Uranium (U(IV) and U(VI)) can form complexes with carbonate, which can increase the extent of the release of U from the soil.

### Effect of HA on U fractions

**Figure 5:**
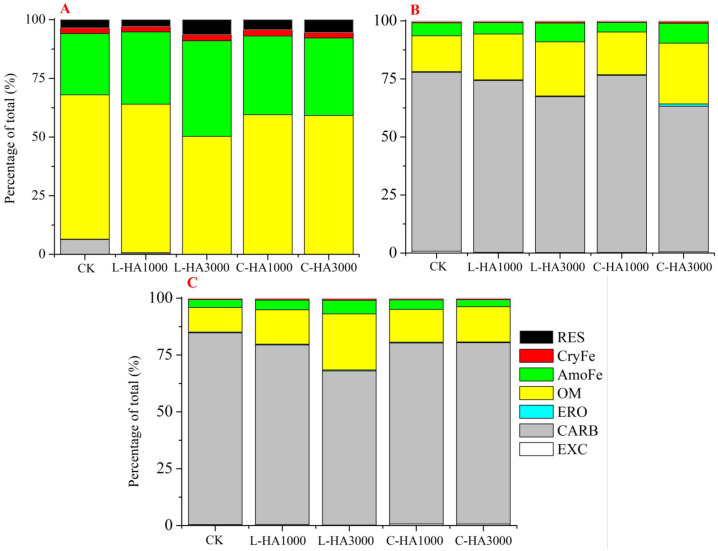
The effect of HA on the redistribution of U fractions after one month incubation. (A) UO_2_, (B) UO_3_, (C) UO_2_(NO_3_)_2_.

The toxicity and mobility of U in the soil can be directly reflected by its fractions and the organic matter (such as HA) is one key factor that controls the U fractions (Bednar et al., 2007; Bone et al., 2019). The effect of HA on the U fractions is shown in Fig. 5, which indicates that HA significantly influences the U fractions in soil. In nature, U is predominantly present as U(VI) under oxidation conditions, and U(IV) can transform into U(VI) in the presence of various cations (such as Ca^2+^, Fe^2+^ or Fe^3+^) and organic matter, such as HA and fulvic acids (FA). These significantly influence the redox reactions associated with uranium (Gu et al., 2005; Stewart et al., 2011). HA containing abundant functional groups (*e.g.*, COOH and phenolic-OH) exhibits a strong propensity toward the adsorption of metals (Meng et al., 2021b). Also, HA can interact with Fe or Mn oxides, clay, and minerals in the soil. Thus, it influences the amount of metal fractions in soil (Tombácz et al., 2004). From Fig. 5, we can observe that the redistribution of U fractions varied with the U and HA sources and the HA content.

When UO_2_ is the source (Fig. 5A), U transforms into different fractions (except the EXC fraction) during the one-month incubation period. The addition of HA decreased the CARB content and increased the content of AmoFe and RES fractions. This indicates that the presence of HA promotes the transformation of uranium into easily soluble fractions (CARB and OM fractions). This results in the formation of intractable soluble fractions (AmoFe and RES fractions). We also observed that the content of the uranium fraction varied with the HA type and dosage. Higher L-HA contents exerted better effects on the process of transformation of U into intractable soluble fractions (especially during the transformation into the AmoFe and RES fractions). For UO_3_ (Fig. 5B), the effects of HA on U fractions were similar. The EXC and CARB fractions decreased with the HA content, while the OM and AmoFe fractions.

Insignificant changes were observed in the other fractions. L-HA exerted different effects on the uranium fraction. An increase in the L-HA content decreased the content of the EXC fraction, while an increase in the C-HA content resulted in an increase in the EXC fraction. This can be attributed to the fact that C-HA is a soluble organic matter with high pH. For UO_2_(NO_3_)_2_ (Fig. 5C), U(VI) is predominantly present in the soil, and the addition of HA had similar effects on the U fractions in the case of UO_2_ and UO_3_ (different observations were made with the EXC fraction). The addition of L-HA resulted in a decrease in the EXC fraction, while the EXC content increased with an increase in the C-HA content. This may be the portion of C-HA that dissolves at a high pH. The effect exerted by L-HA on the transformation of the U fraction (the CARB fraction decreases and the OM and AmoFe fractions increase in content) was greater than the effect exerted by C-HA.

The effects of HA on the transformation of the U fractions can be attributed to various factors. First, the nature of HA has a significant influence on the transformation process. HA with high carbon content and abundant functional groups can form complexes with U (Meng et al., 2021b). Second, HA can interact with soil minerals and Fe-Mn to form a stable organic-minerals combination (Yuan & Theng, 2011). The presence of HA in the soil can improve the adsorption extent of U onto Fe-Mn and minerals. Third, soil pH significantly affects the solubility of HA and the U fractions. In general, the mobility of metal cations increases with a decrease in the pH, and the solubility of HA decreases with a decrease in the pH (Houben, Evrard & Sonnet, 2013; Król, Mizerna & Bozym, 2020). The solubility of HA increases with an increase in pH, increasing the EXC fraction content when UO_3_ and UO_2_(NO_3_)_2_are considered.

## Conclusions

L-HA and C-HA exert different effects on soil pH, the process of U redistribution, and the contents of fractions in the soil. The effects of HA on the process of U redistribution and fraction contents varied with the sources of HA and U. The presence of L-HA results in a reduction in soil pH, while the presence of C-HA increases soil pH. When UO_2_ and UO_3_ are the sources of U, HA can reduce the content of exchangeable and weak-acid soluble U. When UO_2_(NO_3_)_2_ was the source of U, L-HA decreased the content of the exchangeable and weak-acid soluble U, while C-HA exerted the opposite effect. The addition of HA resulted in the easy transformation of the soluble fraction (CARB and OM fractions) into intractable soluble fractions (AmoFe and RES fractions). L-HA exhibits a better potential for use as a remediation material than C-HA.

##  Supplemental Information

10.7717/peerj.14162/supp-1 Supplemental Information 1 The effect of HA on U fractions

10.7717/peerj.14162/supp-2 Supplemental Information 2 Three U species

10.7717/peerj.14162/supp-3 Supplemental Information 3 Soil pH

## Additional Information and Declarations

### Data Availability

The following information was supplied regarding data availability: The raw measurements are available in the Supplementary Files.
